# Increased Dietary Intakes of Total Protein, Animal Protein and White Meat Protein Were Associated with Reduced Bone Loss—A Prospective Analysis Based on Guangzhou Health and Nutrition Cohort, South China

**DOI:** 10.3390/nu15061432

**Published:** 2023-03-16

**Authors:** Zhao-Min Liu, Qi Huang, Huan-Huan Long, Shu-Yi Li, Yi Wu, Su-Juan Zhang, Xin-Yi Tang, Yu-Ming Chen

**Affiliations:** 1 Guangdong Provincial Key Laboratory of Food, Nutrition and Health, Department of Nutrition, School of Public Health, Sun Yat-sen University, Guangzhou 510080, China; 2 Department of Pediatrics, The Third Affiliated Hospital of Sun Yat-sen University, Guangzhou 510630, China; 3 Department of Epidemiology, School of Public Health, Sun Yet-sen University, Guangzhou 510080, China

**Keywords:** dietary protein, animal protein, white meat protein, bone mineral density, amino acids, elderly Chinese

## Abstract

In this study, we aimed to prospectively investigate the relationships between different types of dietary protein and changes in bone mass in Chinese middle-aged and elderly people. Dietary intakes were evaluated by means of a validated food frequency questionnaire. Bone mineral density (BMD) was measured using a dual-energy bone densitometer at multiple bone sites. Multivariable regression models were applied to investigate the associations of the participants’ dietary intakes of total protein, intakes of protein from various sources, and amino acid intakes with the annualized changes in BMD during a 3-year follow-up. A total of 1987 participants aged 60.3 ± 4.9 years were included in the analyses. Multivariable linear regression results showed that dietary intakes of total protein, animal protein, and protein from white meat were positively correlated with BMD changes, with standardized coefficients (β) of 0.104, 0.073, and 0.074 at the femur neck (*p* < 0.01) and 0.118, 0.067, and 0.067 at the trochanter (*p* < 0.01), respectively. With each increase of 0.1g·kg^−1^·d^−1^ in animal protein and white meat protein intakes, the BMD losses were reduced by 5.40 and 9.24 mg/cm^2^ at the femur neck (*p* < 0.05) and 1.11 and 1.84 mg/cm^2^ at the trochanter (*p* < 0.01), respectively. Our prospective data, obtained from Chinese adults, showed that dietary total and animal protein, especially protein from white meat, could significantly reduce bone loss at the femur neck and trochanter.

## 1. Introduction

Osteoporosis (OP) is characterized by low bone mineral density (BMD) and the loss of bone strength, predisposing patients to an increased risk of osteoporotic fractures. With the increase in the aging population, OP and bone fractures have become compelling public health concerns. Statistics from China have shown a prevalence of OP of more than 30% in the elderly (people aged more than 65 years) [1]. Due to its silent nature, OP remains largely undiagnosed and poorly managed, even after the occurrence of a fragility fracture [2]. It has been reported that bone densitometry, the gold standard for OP diagnosis, had never been performed in nearly 70% of fracture patients who were Asian postmenopausal women [2].

Medication treatments for OP are often associated with poor compliance [1,2]. Nutritional factors have been identified as playing an important role in the prevention of OP. Considerable attention has recently been paid to dietary protein as a non-pharmacological approach for maintaining skeletal health in adults. Dietary proteins are as essential as calcium and vitamin D for bone health. This is because collagen and non-collagenous proteins comprise almost 50% of bone volume [3]. The cross-linking of collagen molecules in bone requires the translational modification of amino acids, such as the hydroxylation of lysine and proline [3]. Around one third of bone mass undergoes remodeling [3]. However, unlike vitamin D and calcium, the collagen fragments released during bone remodeling via proteolysis cannot be reutilized for the new bone matrix [4]. Studies have estimated that protein intake accounts for around 2–4% of the BMD variance in adults [3]. Among the factors that affect skeletal strength and integrity, dietary protein remains one of the most controversial [3,5]. This is due to conflicting factors which mean that, at least in theory, protein may exert both beneficial and detrimental effects on bone [6].

Several systematic reviews and meta-analyses [5,7,8,9] have been conducted to summarize the association of dietary protein intake with bone features; however, these findings are still inconclusive and their validity is limited due to the heterogeneity of the included studies. The diversity in study design and duration, population features, protein dosages and sources, outcome measures, calcium adequacy, and uncontrolled confounders have been the essential drivers of heterogeneity. Excess protein intake was reported to be deleterious to bone health via an increase in the diet-derived acid load [10,11], especially in diets rich in animal foods and low in fruits and vegetables, or with insufficient calcium intake. The magnitude of this detrimental effect might be more evident in the elderly due to the aging-related decrease in the renal ability to excrete acid loads. Although observational studies and randomized controlled trials have been conducted to address the effectiveness of protein intake on bone health [5,7,8,9], most of these studies were performed in Western populations. Differences in genetic predisposition, dietary habits, and lifestyles could affect these results. Due to the spread of urbanized lifestyles, the Chinese elderly population has adopted more sedentary lifestyles, less physical work, and more Westernized dietary patterns in this transition period. In addition, longitudinal studies comparing the effects of various types of dietary protein (i.e., animal vs. plant proteins, red meat vs. white meat protein), as well as amino acid profiles, on bone mass are scarce [5]. We thus proposed to conduct analyses based on a cohort study in a middle-aged and elderly Chinese population to examine the associations of total dietary protein, different sources of protein, and amino acid profiles with the changes in BMD during a 3 year follow-up.

## 2. Methodology

The analyses were based on a prospective cohort study, the Guangzhou Nutrition and Health Study (GNHS, https://clinicaltrials.gov, No. NCT03179657). The study protocol was approved by the Ethics Committee of the School of Public Health, Sun Yat-sen University. All of the participants signed informed consent forms before enrollment. Baseline data were collected from 2008 to 2010, with follow-ups made every 3 years after that. The details of the recruitment process have been reported previously [12]. In brief, participants aged 40–75 years who had resided in Guangzhou for at least 5 years were recruited through advertisements or referrals. They were excluded if they exhibited cognitive impairment or mental disorders, poor vision or blindness, severe hepatic or renal dysfunction, or were unable to walk. A total of 3169 eligible men and women were recruited at baseline, among which 2496 and 2179 attended the first (2011 to 2013) and second follow-up (2014 to 2017) visits, respectively. The analyses reported here were conducted on the basis of these two follow-ups. Participants were required to complete a questionnaire survey and BMD measurement during both follow-ups. We excluded 192 participants from the analysis for having any of the following conditions: a medical history of serious chronic diseases, including malignancies, clinical hyperthyroidism, or type 2 diabetes; having ever used medications for OP; currently taking certain medications such as glucocorticoids, thyroid hormones, diuretics, and isoniazid; and having a total energy intake > 5000 kcal/d or <500 kcal/d.

### 2.1. Questionnaire Survey and Anthropometric Measures

The questionnaire survey was conducted by trained investigators via face-to-face interviews to collect information on socioeconomics and demographics, lifestyle factors (i.e., smoking, alcohol, tea and coffee drinking, habitual dietary intakes, and physical activities), family and medical history, and supplement usage. Habitual physical activities in the preceding month were investigated, and energy expenditure for total activities and different types of activities (occupation, leisure time, household, and sports) were estimated. Participants’ body weights were measured without shoes on a balance-beam scale, and height was measured with a Harpenden stadiometer. The body mass index (BMI, in kg/m^2^) was then calculated.

### 2.2. Dietary Assessment

Dietary intakes were investigated at the 1st follow-up using a validated 79-item food frequency questionnaire (FFQ) [13] to estimate habitual food intakes during the preceding year. For each food item, its frequency (never or per year, month, week, or day) of consumption and the regular serving size were estimated. Colorful pictures of foods in standard sizes were provided to help reduce the recall bias of participants. Commonly used portion sizes (bowl, glass, slice, or other utensil units) were specified for each food item. The dietary intake of total energy, protein, amino acids, and other nutrients was calculated according to the Chinese Food Composition Table 2009 [14]. Nutrients were adjusted for total energy using the residual method as appropriate. The intakes of total protein and protein from various sources (i.e., animal and plant protein, red and white meat protein) were estimated as grams per day (g/d), grams per kilogram of body weight (g/kg.BW), and the percentages of total energy intake (% energy). Daily intakes of nutrients were determined by multiplying the daily amount of individual food items by their respective content in foods and then adding them together. Animal proteins were proteins from red meat (pork, beef, lamb, etc.), white meat (poultry, fish, seafood, etc.), and others (eggs and dairy products, etc.). Plant protein included proteins from vegetables, fruits, nuts, soy, other legumes, and grains. We also estimated the dietary intakes of major essential amino acids (AA) and sulfur amino acids (methionine and cysteine).

The validity and reproducibility of the FFQ have been tested among a local population (*n* = 61) using a total of 6×3 days of dietary records (DRs) acquired over one year [13], of which the relative validity assessed using Spearman correlation coefficients was 0.32–0.38 for nutrients and 0.31–0.56 for food groups. The adjusted correlation coefficients were 0.52 (between two FFQs) and 0.46 (between the FFQ and DRs) for protein intake, 0.66 and 0.48 for dairy products, and 0.39 to 0.59 for energy-adjusted protein-rich foods (egg, dairy products, meat, soy and fish, etc.). The misclassification rate of participants into opposite quartiles in relation to protein intake was only 3%.

### 2.3. Bone Mineral Density (BMD) Measurements

During the two follow-ups, dual-energy X-ray absorptiometry (DXA, Hologic QDR-1000, version 6.10; Hologic, Inc., Waltham, MA, USA) was used to measure the BMD (mg/cm^2^) at multiple bone sites, including the whole body (WB), lumber spine (LS, L_1_–L_4_), total hip (TH), femur neck (FN)m and trochanter using Hologic Discovery software, version 3.2. The intra-coefficients of variation of the duplicated BMD measurements in 30 participants after re-positioning were 1.18% (WB), 0.87% (LS), 1.02% (TH), and 1.92% (FN), respectively. The annualized change in BMD between the two follow-ups was calculated as follows: (BMD _follow-up 2_ − BMD _follow-up 1_) × 3 years / intervals of two follow-ups.

### 2.4. Statistical Analysis

Statistical analyses were conducted using IBM Statistic SPSS software (version 22.0) with the significance established at two-sided *p* values < 0.05. Analysis of variance (ANOVA) and chi-squared tests were used to compare the differences among groups in terms of continuous and categorical variables, respectively. There were no significant interactions between dietary total protein intake and gender in regard to BMD changes; thus, all results were reported without separation by gender. Multivariable linear regression was applied to investigate the associations of total dietary protein, different protein sources (animal and plant protein, red and white meat protein, soy protein), and dietary amino acid intakes with the annualized changes in BMD. Analyses of covariance (ANCOVA) were undertaken to compare BMD changes by quartiles of total protein intakes and intakes from various sources of protein (Q1–Q4), with the lowest quartile as the reference group. Potential confounders were introduced by means of the enter method and determined according to the univariate results, biological background, and a literature review. Models were adjusted for age, gender, body mass index, marital status, household income, smoking, alcohol drinking, physical activity, calcium supplement usage, dietary energy intake, vitamin D, and calcium intake. Trend tests were conducted by including quartile numbers as continuous variables.

Subgroup analyses were conducted to explore the effect modifications by gender, different age groups (<60 vs. ≥60, y), BMI (<24 vs. ≥24, kg/m^2^), and dietary calcium intake (<400 vs. ≥400, mg/d). Interactions between quartiles of total protein intake with subgroup variables were tested before the stratification of data by the inclusion of the production term in the regression models. Substitution modeling by means of the leave-one-out method was performed to estimate the impact of replacing red meat protein with white meat protein. This model included the variables of white meat protein intake, animal protein intake, and other sources of animal protein (egg and dairy protein, etc.) but not red meat protein intake in the multivariable linear regression model, with the adjustment of possible confounders.

## 3. Results

### Participants’ Characteristics in the First and Second Trimesters

**Figure 1:**
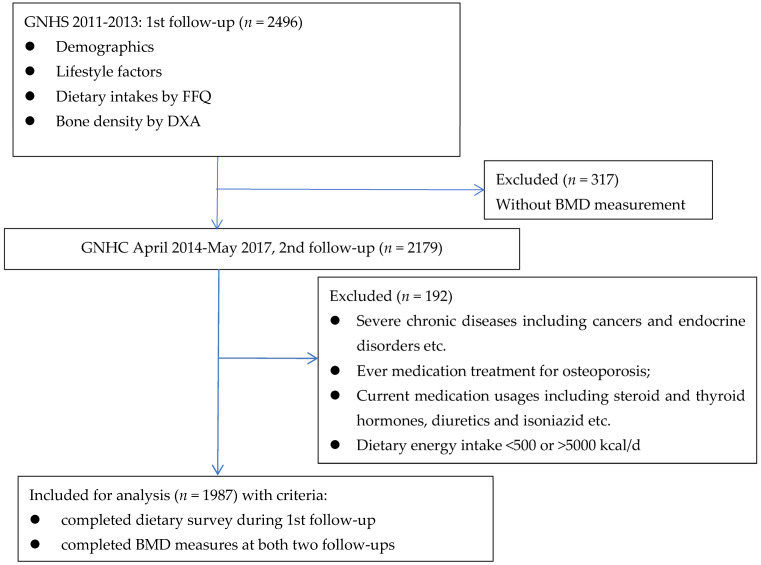
Study flowchart. Abbreviations: GNHS, Guangzhou Nutrition and Health Study; FFQ, food frequency questionnaire; DXA, dual-energy X-ray absorptiometry; BMD, bone mineral density.

A total of 1987 participants who had completed the dietary survey at the first follow-up and BMD measurements at both the 1st and 2nd follow-ups were included for analysis. The details of enrollment are indicated in Figure 1. The average interval between two follow-ups was 2.37 years. Participants’ characteristics and dietary intakes at the 1st follow-up are presented as quartiles of total dietary protein intakes in Table 1 and Supplemental Table S1. The mean age of the participants was 60.3 ± 4.9 (y), with a mean BMI of 23.5 ± 3.2 (kg/m^2^). Dietary total protein accounted for 17.5% ± 5.8% of total energy, among which 49.1% was from animal foods. The daily average protein intakes (g·kg^−1^·d^−1^) were 1.13 ± 0.24 for total protein, 0.57 ± 0.21 for animal protein, 0.57 ± 0.15 for plant protein, 0.22 ± 0.13 for red meat protein, and 0.21 ± 0.13 for white meat protein. The highest quartile group had a significantly higher energy expenditure for physical activity and a higher dietary calcium intake than the lowest quartile of total protein intake. Compared with the 1st follow-up, BMD changes at the 2nd follow-up were lower in the whole body, total hip, and femur neck categories, whereas they were increased in the spine L_1_ category.

**Figure 2:**
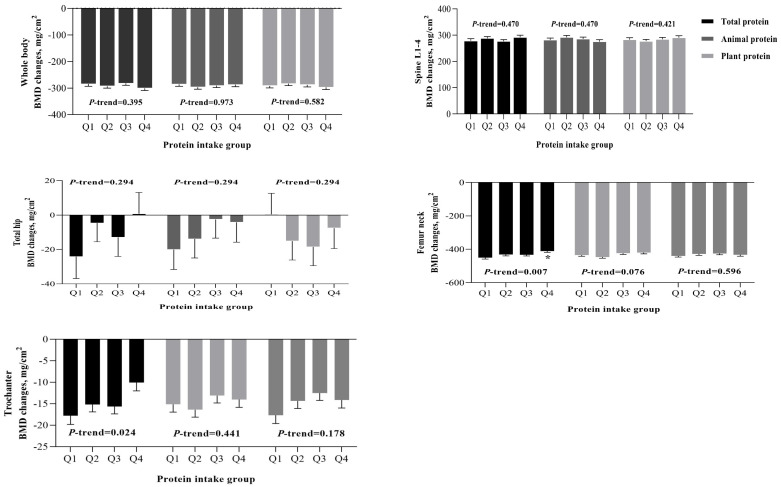
Changes in bone mineral density (BMD) at multiple bone sites by quartiles (Q1 to Q4) of dietary intakes of total protein, animal and plant protein. Models were adjusted for age, gender, body mass index, marital status, household income, smoking, alcohol drinking, physical activity, calcium supplement usage, dietary energy intake, dietary vitamin D intake, and calcium intake. Trend analysis was conducted by including quartile ordinal numbers as a variable in the general linear model. * *p* < 0.05.

Multivariable linear regression was used to test the relationships of various dietary protein intakes with the annualized BMD changes during a 3-year follow-up (Table 2 and Figure 2). After controlling for potential confounders, significant or marginally significant and positive associations were observed between dietary intakes of total protein (standardized coefficient, *β* = 0.104 and 0.118, *p* < 0.001), animal protein (*β* = 0.073 and 0.067, *p* < 0.05), and protein from white meat (*β* = 0.074 and 0.067, *p* < 0.01) with changes in BMD at the femur neck and trochanter, respectively. No significant associations were observed in other bone sites.

When dietary protein intakes were further treated as quartiles (Table 3), after the full adjustment of covariates (model 2), compared with the lowest quartile, participants in the highest quartile of total protein had significantly lowered bone losses, with the change% of 9.48% (*p* = 0.012) at the femur neck (*P_trend_* = 0.003) and 46.67% (*p* = 0.049) at the trochanter (*P_trend_* = 0.018). Only a marginal benefit from animal protein intake was observed at the femur neck, with a reduced bone loss of 3.18% (*p* = 0.057) when comparing the extreme quartiles (*P_trend_* = 0.084) (Table 4). No significant association was observed between plant protein intakes and BMD changes (Supplemental Table S2). After adjusting for potential confounders, white meat protein intake resulted in reduced bone losses of 7.16% at the femur neck (*p* = 0.030) and 31.21% at the trochanter (*p* = 0.122) with a notable dose-response relationship (both *P_tren__d_* < 0.05) (Table 5). Multivariable linear regression analysis indicated that (Table 6), with the exception of lysine and histidine, other dietary amino acids were positively related to BMD changes at either the femur neck or trochanter, with regression coefficients ranging from 0.12 to 3.82 (*p* < 0.05).

Subgroup analyses (Supplemental Tables S3–S6) based on the variables of gender, age, BMI, and dietary calcium intake suggested similar findings with the whole participants. Still, the associations seemed more evident among women and individuals of normal BMI (18.5–24.0, kg/m^2^). For participants with a normal BMI, total protein, especially red-meat protein, even displayed an adverse effect on BMD changes at the whole-body level. Substitution modeling (Supplemental Table S7), replacing red meat protein with an equivalent amount of white meat protein in the multivariate linear regression model, indicated increased values of coefficients between white meat protein intakes and the changes in BMD in the whole body (from 22.9 ± 40.3 to 33. 7 ± 44.6, mg/cm^2^), the femur neck (from 64.5 ± 38.8 to 82.9 ± 42.9, mg/cm^2^), and the trochanter (from 13.9 ± 9.5 to 15.9 ± 10.5, mg/cm^2^).

## 4. Discussion

### 4.1. Summary of Current Findings

This longitudinal study among a middle-aged and elderly Chinese population showed that higher levels of consumption of total and animal protein and protein from white meat were associated with significantly reduced bone losses at the femoral neck and trochanter. Favorable linkages were not observed with red meat protein or plant protein. The bone-protective effect of protein was more evident in women and participants with normal BMIs. To our knowledge, this is the first study that has reported the favorable impact of white meat protein on the alleviation of bone loss in the Chinese population. We were able to detect these beneficial changes in bone mass even in a relatively short follow-up period among a less aged population. Our findings indicate that the beneficial impact of total protein on bone mass might be mainly attributable to white meat protein. A dietary regimen for elderly Chinese adults containing a recommendation of the consumption of major animal protein sourced from white meat would be of essential clinical and public health value in preventing aging-related bone loss and decreasing healthcare costs. Based on a recent report on postmenopausal women in the US, which showed that each decrease of 1 SD in femoral neck BMD (11.7 mg/cm^2^) was associated with a 1.8-fold increased risk of hip fracture over 9 years [15], considering our findings we may expect a reduction in hip fracture risk of 16% or 27% with a daily increase of 10 g total protein or white meat protein, on the basis of the annual BMD changes at the femur neck in our data, assuming a body weight of 60 kg. The findings could also assist in designing trials involving the substitution of red meat protein with white meat protein in populations at high risk of bone loss.

### 4.2. Protein Intake among the Elderly Chinese Population

An adequate protein supply, appropriate protein sources, and an appropriate amino acid composition are essential for the optimal acquisition and maintenance of the muscular-skeletal system. However, the current recommendations for dietary protein intake were largely established on the basis of non-bone health outcomes and they vary among different ethnic populations and physiological stages [16]. Higher intakes of 1.0–1.2 g·kg^−1^·d^−1^ have been proposed for the elderly due to the attenuated anabolic response that occurs with aging [4], and even 1.2–1.5 g·kg^−1^·d^−1^ for elderly people with acute or chronic illness [17]. Malnutrition of protein (<0.8 g·kg^−1^·d^−1^) not only causes the release of calcium from bone but may also increase the rate of falls due to impaired muscle strength, coordination, and reaction time, which could increase the risk of fractures [18]. An insufficient dietary protein intake may be a much more severe problem than an excess of protein [3]. The daily total protein intake of our participants was 1.13 g·kg^−1^·d^−1^, with 91.4% (88.6% for men and 92.6% for women) achieving the WHO recommendation of 0.83 g·kg^−1^·d^−1^ [19], and 77.8% (69.1% for men and 81.3% for women) exceeding the recommendations of the Chinese Nutritional Society of 65 g/d for men and 55 g/d for women (≥65 y). Animal-sourced protein accounted for 49.1% of total protein, among which 38.6% was obtained from red meat and 36.8% from white meat. Our data implicated that, although the total protein intake was adequate in most of our participants, there was still sufficient room for the improvement of protein sources by increasing white meat intake to promote bone health. Our substitution analysis further indicated that when replacing red meat with white meat protein in an equivalent protein amount, an additional benefit of lowered bone loss could be expected.

The role of protein intake in relation to bone mass and fracture risk has been controversial. Several large observational studies have revealed the benefits of higher protein intakes in attenuating age-related bone loss or reducing fracture risk [3,5,7,20,21], whereas others have reported negative or even adverse findings [8,22,23]. Differences in study designs, population features, nutritional status with regard to calcium, weight changes, and lifestyle factors might all lead to discordant associations [9,23]. A cross-sectional study [21] based on UK biobank data, including 70,215 adult men and women, indicated that higher total protein intakes were positively associated with the ultrasound-evaluated bone mass of the heel. Another study, conducted among postmenopausal women, also reported that a 20% increase in energy-calibrated protein intake was associated with a significantly higher BMD for the total body and hip, which was associated with a 7% lower fracture risk in the forearm over a 12-year follow-up [20]. The possible mechanisms for these effects could be related to the observation that protein intake affects bones not only by providing the structural matrixes of bones via regulating signaling transduction, i.e., the hormone/insulin-like growth factor (GH/IGF) axis and stimulating the secretion of IGF-1 and a decrease in parathyroid hormone (PTH), but also by increasing intestinal calcium absorption, reducing bone turnover marker levels, or enhancing lean body mass [24].

Protein from different sources has been inconsistently demonstrated to aid bone health. Our longitudinal findings are in line with several previous reports [25,26,27,28] showing that animal protein but not plant protein could alleviate bone loss or reduce fracture risks, but not all [5,23]. The relative superiority of animal protein over plant protein may be related to the calcium contained in animal foods, with good absorption and bioavailability; high levels of aromatic AA in animal products [29]; other bone-favorable components in the diet (seafoods and dairy products); or dietary patterns [6,30]. Early studies revealed that a high protein intake, especially of protein from animal sources, could increase the acid load, which resulted in the release of calcium from bones or induced a shift in systemic pH to increase osteoclastic bone resorption, but this hypotheses seemed untenable in subsequent studies due to the strong buffering system of the human body [6,31]. Furthermore, an increased acid load can be contradicted by increasing calcium absorption to achieve balanced calcium retention, or through the alkalinizing effects of fruits and vegetables [6]. Compared with other reports, our findings indicated that significant changes in bone mass due to the intake of various sources of protein were only observed at the femoral neck and trochanter, which could be due to the higher composition of the trabecular bone and the lower levels of volumetric BMD compared to other sites, or the expansion of the bone diameter with aging, leading to thinner walls and lower bone density [32].

The favorable impact of protein intake, which was manifested as a decrease in bone loss, could be mediated by dietary amino acids. Different amino acids may affect various processes related to protein metabolism in bones by regulating the proteasome depending on proteolysis and protein synthesis, or modifying immune function [33]. An appropriate amino acid profile is essential to support bone remodeling and formation through increasing calcium re-absorption and IGF-1 production or decreasing serum PTH and calcitriol levels [34]. A cohort study revealed that a specific AA profile, evaluated based on circulating AAs, was correlated with greater BMD and a lower subsequent fracture risk, independently of diet and lifestyle factors [35]. Sulfur amino acids are mainly present in animal-derived proteins and are reported to be positively correlated with bone mass [36]. Methionine has been shown to improve cartilage formation and decrease the degree of osteoclast development in blood mononuclear cells in OVX rats [37]. Phenylalanine could be a signal molecule which increases the flow of calcium into the cell and reduces calcium loss. Tryptophan is the major component or substrate of melatonin, which can reduce TNF-α levels and enhance bone formation [38]. Two metabolomics studies also revealed that serum concentrations of cysteine were positively associated with bone mass in Caucasian women [39] and postmenopausal women [40].

### 4.3. Mechanisms of White Meat Protein and the Slowing Down of Bone Loss

Our analysis indicated that a favorable effect of total dietary protein on bone could be attributable to white meat protein. Further substitution analysis suggested that white meat protein, rather than red meat protein, would exert more beneficial impacts by slowing down bone loss. Previous studies have reported that white meat consumption, compared with the consumption of red meat or processed meat, was associated with a decreased risk of cardio-metabolic disorders, cancers, and mortality [41]. However, studies linking total white meat consumption with bone health are rare, although some investigations have reported that the intake of fish or seafood [42,43] or a Mediterranean dietary pattern [44,45] were beneficial to bone health, but not all showed beneficial associations [46,47]. A study by Choi and Park [48] revealed a weak positive association with BMD in Koreans who consumed seafood 23 times/month on average but not in Americans who consumed seafood only five times/month, suggesting that a minimum seafood intake might be necessary for a significant effect on bone status.

The biological mechanisms of the favorable association of white meat consumption with the maintenance of bone mass might be related to the abundance of polyunsaturated fatty acids (PUFAs) in poultry and fish [49]; the particular amino acid profile, consisting of high levels of isoleucine, threonine, and valine, which can increase IGF-1 but inhibit IL-1 and TNF synthesis [50]; or a large number of antioxidants such as di- and oligopeptides and the imidazole moiety [51]. In addition, in contrast to red and processed meat, white meat contains less heme iron [43], which could contribute to the suppression of an increase in the endogenous formation of N-nitroso compounds (NOCs) [52]. The nitrites and nitrates in red meat can lead to elevated oxidative stress, which may harm bone health [53]. An increase in white meat consumption may imply a relative reduction in red meat. A recent metabolomics study indicated that the consumption of red and processed meat results in higher levels of short- and medium-chain acylcarnitines and 3-dehydroxycarnitine [54], which is related to OP. Red and processed meat could also contribute to adverse metabolic profiles and adversely affect bone health [55].

Our subgroup findings indicated that the beneficial association of protein intake with bone mass was more evident among participants with normal BMIs. Although excess body fat exerts mechanical overload on the bones, obesity can also result in metabolic disarrangements, such as hyperinsulinemia or disturbance of the absorption of bone-beneficial nutrients [56], which may negatively affect the bones. In addition, the mechanisms related to the dysfunction of bone-regulating hormones have been shown to decrease calcium absorption and low-grade systemic inflammation may explain the adverse effect of fat mass, especially that of visceral adipose tissue, on bone cell metabolism, which could compromise the quality of bone mass [57].

### 4.4. Study Strengths and Limitations

Our study has several advantages, such as the longitudinal study design, the relatively large sample size for BMD measurements, the availability of validated dietary data, extensive covariates for the adjustment of confounders, and annualized BMD changes as outcome measures. We assessed the association of protein intakes with BMD at various bone sites and comprehensively investigated the protein intakes in terms of the adequacy of the amounts, different sources and subtypes of proteins, as well as amino acid profiles. The substitution analysis provided novel evidence on the prediction of a BMD improvement afforded by replacing red meat with white meat protein, which extended the acknowledged relationship between total dietary protein levels and bone.

The limitations of this study should also be acknowledged. First, FFQ may suffer from measurement errors due to recall bias, although it has been validated. The quantification of protein consumption through a dietary survey may have led to misclassification. However, it was likely to be non-differential and only attenuated the possible associations. Although serum urea was identified as an accepted index of dietary protein intake, 24 h urine collection for the analysis of urea excretion is restricted in large-scale epidemiological studies. Second, although we observed that animal protein, especially white meat consumption, was associated with improved bone mass, we did not consider food processing methods. The consumption of processed meat could increase the risk of chronic disorders. Third, although in our regression models we adjusted for a range of risk factors, the influence of residual confounding factors could not be completely excluded. In addition, participants who took part in an OP study may have had a heightened awareness of their bone health. However, it seems unlikely that this would influence protein consumption because protein is not commonly perceived as an OP risk factor, and the current findings on this matter are still debatable. Lastly, we did not follow up on patients for the occurrence of fractures. Additionally, in our study, we only investigated different sources of protein and amino acid profiles, without considering their metabolites, which may hinder mechanistically relevant pathway analyses, which are possibly achievable in metabolomics studies.

## 5. Conclusions

This longitudinal study on middle-aged and elderly Chinese people indicated that higher intakes of total protein and animal protein, especially white meat protein, were associated with attenuated bone loss. Given the increasing prevalence of OP and sarcopenia, further studies are needed to determine the optimal amount of protein for muscular-skeletal health in elderly people. The beneficial effect of white meat protein should be further confirmed in more prospective studies and tested in randomized controlled trials.

## Figures and Tables

**Table 1:** Participants’ characteristics, dietary intakes, and bone mineral density at the 1st follow-up (2011 to 2013) expressed in quartiles of total protein intakes, Guangzhou Nutrition and Health Study (*n* = 1987).

	Total Protein Intakes (g·kg^−1^·d^−1^)					
	Q1 (<0.96)	Q2 (0.96~)	Q3 (1.10~)	Q4 (≥1.26)	Total	*p*
n	497	497	497	496	1987	
Age, year	60.3 ± 5.0	60.4 ± 4.7	60.3 ± 4.8	60.1 ± 5.1	60.3 ± 4.9	0.757
Women, n (%)	327 (65.8)	347 (69.8)	359 (72.2)	391 (78.8)	1424 (71.7)	<0.001
BMI, kg/m^2^	26.1 ± 3.1	24.1 ± 2.4	22.8 ± 2.3	21.1 ± 2.4	23.5 ± 3.2	<0.001
Income ≤ 3000 Yuan·month^−1^·person^−1^, n (%)	288 (58.3)	268 (54.1)	277 (56.1)	309 (62.3)	1142 (57.5)	0.126
Married, n (%)	447 (89.9)	452 (91.1)	433 (87.1)	427 (86.1)	1759 (88.5)	0.043
Smoking, n (%)	58 (11.7)	37 (7.4)	40 (8.0)	30 (6.0)	165 (8.3)	0.011
Habitual alcohol drinking, n (%)	41 (8.2)	35 (7.0)	36 (7.2)	33 (6.7)	145 (7.3)	0.796
Habitual tea drinking, n (%)	299 (60.2)	274 (55.1)	262 (52.7)	248 (50.0)	1083 (54.5)	0.011
Calcium supplements, n (%)	150 (30.2)	138 (27.8)	153 (30.8)	152 (30.6)	593 (29.8)	0.716
Medical history						
Hypertension, n (%)	146 (29.4)	122 (24.6)	126 (25.5)	112 (22.6)	506 (25.5)	0.094
Stroke, n (%)	10 (2.0)	8 (1.6)	9 (1.8)	9 (1.8)	36 (1.8)	0.973
Hyperlipidemia, n (%)	192 (38.7)	207 (41.7)	188 (38.0)	191 (38.6)	778 (39.3)	0.621
HRT, n (%) women only	14 (4.3)	27 (7.8)	20 (5.6)	33 (8.4)	94 (6.6)	0.095
Multivitamin usage, n (%)	85 (17.1)	87 (17.5)	111 (22.3)	109 (22.0)	392 (19.7)	0.059
Total physical activity, Mets/week	24.1 ± 6.1	25.3 ± 6.4	25.2 ± 6.4	25.7 ± 6.8	25.1 ± 6.5	0.001
Dietary intakes						
Total energy, kcal/d	1663.8 ± 496.9	1595.1 ± 456.2	1629.5 ± 460.2	1591.8 ± 531.8	1620.1 ± 487.7	0.067
Fat, % total energy	31.2 ± 14.4	32.1 ± 16.3	31.6 ± 14.8	32.5 ± 15.6	31.9 ± 15.3	0.552
Carbohydrate, % total energy	57.6 ± 22.1	59.1 ± 25.4	58.3 ± 24.9	61.2 ± 25.8	59.1 ± 24.6	0.110
Total protein, % total energy	14.9 ± 4.7	16.9 ± 4.9	17.8 ± 5.5	20.3 ± 6.6	17.5 ± 5.8	<0.001
Animal protein, % total protein	43.59 ± 11.26	47.93 ± 10.85	50.79 ± 10.92	54.22 ± 11.12	49.13 ± 11.70	<0.001
Calcium, mg/d	447.1 ± 129.1	510.1 ± 147.1	529.1 ± 153.1	564.3 ± 183.0	512.3 ± 159.7	<0.001
Vitamin D, μg/d	3.44 ± 2.20	3.75 ± 2.29	4.16 ± 3.20	4.08 ± 2.88	3.86 ± 2.69	<0.001
Total protein, g·kg^−1^·d^−1^	0.85 ± 0.09	1.04 ± 0.04	1.19 ± 0.05	1.46 ± 0.17	1.13 ± 0.24	<0.001
Animal protein, g·kg^−1^·d^−1^	0.37 ± 0.11	0.50 ± 0.12	0.60 ± 0.13	0.80 ± 0.21	0.57 ± 0.21	<0.001
Red-meat protein, g·kg^−1^·d^−1^	0.15 ± 0.08	0.19 ± 0.09	0.23 ± 0.11	0.30 ± 0.17	0.22 ± 0.13	<0.001
White-meat protein, g·kg^−1^·d^−1^	0.13 ± 0.07	0.18 ± 0.08	0.22 ± 0.10	0.32 ± 0.17	0.21 ± 0.13	<0.001
Milk and dairy protein, g·kg^−1^·d^−1^	0.05 ± 0.04	0.07 ± 0.06	0.08 ± 0.07	0.09 ± 0.09	0.07 ± 0.07	<0.001
Egg protein, g·kg^−1^·d^−1^	0.05 ± 0.03	0.06 ± 0.04	0.07 ± 0.05	0.09 ± 0.06	0.07 ± 0.05	<0.001
Plant protein, g·kg^−1^·d^−1^	0.48 ± 0.10	0.54 ± 0.11	0.58 ± 0.13	0.66 ± 0.16	0.57 ± 0.15	<0.001
Soy protein, g·kg^−1^·d^−1^	0.05 ± 0.04	0.06 ± 0.05	0.08 ± 0.07	0.11 ± 0.11	0.07 ± 0.08	<0.001
Changes of BMD, 3 mg/cm^2^						
Whole body	−262.4 ± 145.0	−286.7 ± 148.0	−287.9 ± 207.7	−323.4 ± 151.1	−290.1 ± 166.3	<0.001
Spine L_1_–L_4_	262.4 ± 152.1	284.6 ± 148.6	281.8 ± 164.0	307.0 ± 152.0	283.9 ± 155.0	<0.001
Total hip	−40.4 ± 252.1	−7.0 ± 251.7	−8.7 ± 233.2	16.4 ± 225.3	−10.0 ± 241.5	0.003
Femur neck	−456.8 ± 155.4	−434.8 ± 160.7	−431.4 ± 177.6	−403.0 ± 136.5	−431.5 ± 159.3	<0.001
Trochanter	−15.9 ± 36.6	−14.7 ± 35.1	−15.9 ± 48.8	−12.1 ± 30.9	−14. 7 ± 38. 5	0.356

Data are presented as mean ± SD for continuous variables and compared via analysis of variance (ANOVA) or n (%) for categorical variables and compared by means of χ^2^ tests. Abbreviations: BMI, body mass index; HRT, hormone replacement therapy; METs, metabolic equivalents with unit of kcal/min/kg.BW. Dietary nutrient intakes were estimated based on the Chinese Food Composition Table. Dietary nutrient intakes were residually adjusted by adding residuals to the mean energy intakes of participants. Smoking was defined as active smoking of an accumulated total of more than 5 packages of cigarettes per year. Habitual alcohol drinking was defined as drinking alcohol at least 1 time per week for more than 6 months; habitual tea drinking was defined as at least 2 times per week for more than 6 weeks. Changes in BMD = [(BMD _follow-up 2_ − BMD _follow-up 1_) × 3]/time interval. Red meat protein included meat protein from pork, beef, and lamb. White meat protein included meat protein from poultry, fish, and seafood.

**Table 2:** Associations of dietary protein intakes with the changes in bone mineral density (mg/cm^2^) during follow-ups (n = 1987).

	Changes in Bone Mineral Density (mg/cm^2^)									
Protein Intakes, g·kg^−1^·d^−1^	Whole Body		Spine L_1_–L_4_		Total Hip		Femur Neck		Trochanter	
	B ± SE	β	B ± SE	β	B ± SE	β	B ± SE	β	B ± SE	β
Total protein	−4.71 ± 20.86	−0.007	8.54 ± 19.21	0.014	31.28 ± 30.51	0.032	67.79 ± 20.07 ***	0.104	18.73 ± 4.90 ***	0.118
Animal protein	11.11 ± 20.12	0.014	−7.99 ± 18.52	−0.011	34.66 ± 29.44	0.031	53.61 ± 19.37 **	0.073	12.05 ± 4.73 *	0.067
Red-meat protein	−5.09 ± 30.46	−0.004	12.90 ± 28.04	0.011	29.06 ± 44.61	0.016	18.16 ± 29.38	0.015	5.85 ± 7.18	0.020
White−meat protein	23.89 ± 30.16	0.019	−30.61 ± 27.77	−0.026	65.55 ± 44.26	0.036	89.44 ± 29.03 **	0.074	19.77 ± 7.10 **	0.067
Milk and dairy protein	61.19 ± 80.67	0.025	−25.16 ± 74.28	−0.011	−85.04 ± 117.87	−0.024	76.60 ± 77.82	0.033	12.29 ± 19.01	0.022
Egg protein	−20.46 ± 86.76	−0.006	28.92 ± 79.88	0.009	−31.51 ± 126.90	−0.006	21.43 ± 83.71	0.006	−1.01 ± 20.45	−0.001
Plant protein	−40.51 ± 32.53	−0.035	41.69 ± 29.95	0.039	−14.45 ± 47.64	−0.009	24.67 ± 31.39	0.022	14.04 ± 7.66	0.052
Soy protein	−47.73 ± 52.49	−0.022	19.27 ± 48.34	0.009	93.98 ± 76.79	0.029	−28.07 ± 50.65	−0.013	9.03 ± 12.37	0.018

Data are presented as both regression coefficients ± standard error (B ± SE) and standardized coefficients (β). The analyses were undertaken via multivariable linear regression by means of the enter method with adjusted covariates including age (y), sex (male or female), BMI (kg/m^2^), marital status (yes or no), household income (≤3000, 3000–6000 or >6000 yuan·month^−1^·person^−1^), smoking status (yes or no), drinking status (yes or no), total physical activity (Met/week), calcium supplement usage (yes or no), multi-vitamin usage (yes or no), dietary energy intake (kcal/d), dietary vitamin D intake (μg/d), and calcium intake (mg/d). Changes in BMD = [(BMD _follow-up 2_ − BMD _follow-up 1_) × 3]/time interval. Red meat protein included meat protein from pork, beef, and lamb. White meat protein included meat protein from poultry, fish, and seafoods. * *p* < 0.05, ** *p* < 0.01, *** *p* < 0.001.

**Table 3:** Changes in bone mineral density (BMD) (mg/cm^2^) between two follow-ups by quartiles (Q) of total protein intakes (n = 1987).

	Total Protein Intakes (g·kg^−1^·d^−1^)						
	Q1 (<0.96)	Q2 (0.96~)	Q3 (1.10~)	Q4 (≥1.26)	% Difference	*p*-ANCOVA	*p* Trend
	n = 497	n = 497	n = 497	n = 496			
BMD changes, mg/cm^2^							
Whole body							
Model 1	−287.79 ± 8.35	−291.13 ± 7.47	−280.25 ± 7.55	−295.67 ± 8.30	−2.74	0.531	0.756
Model 2	−283.36 ± 8.81	−291.61 ± 7.57	−281.66 ± 7.64	−298.60 ± 8.59	−5.38	0.435	0.416
Spine L1-4							
Model 1	282.11 ± 7.72	287.18 ± 6.91	275.37 ± 6.98	289.24 ± 7.68	2.53	0.503	0.801
Model 2	279.19 ± 8.12	287.18 ± 6.97	275.85 ± 7.03	290.74 ± 7.91	4.14	0.443	0.565
Total hip							
Model 1	−23.77 ± 12.13	−4.88 ± 10.99	−12.36 ± 11.13	0.24 ± 12.21	101.01	0.561	0.285
Model 2	−27.29 ± 12.89	−7.14 ± 11.07	−11.52 ± 11.24	2.52 ± 12.33	109.23	0.485	0.187
Femur neck							
Model 1	−445.31 ± 8.07	−428.32 ± 7.22	−432.53 ± 7.30	−410.37 ± 8.03 *	7.85	0.037	0.011
Model 2	−449.67 ± 8.49	−427.74 ± 7.28	−431.23 ± 7.35	−407.05 ± 8.27 **	9.48	0.012	0.003
Trochanter							
Model 1	−17.39 ± 1.97	−14.73 ± 1.76	−15.66 ± 1.78	−10.13 ± 1.96	41.74	0.083	0.033
Model 2	−18.02 ± 2.08	−14.75 ± 1.78	−15.55 ± 1.80	−9.61 ± 2.02	46.67	0.049	0.018

Data are presented as the adjusted mean ± standard error based on the general linear model, with the Q1 as the reference group. * *p* < 0.05, ** *p* < 0.01. Model 1 was adjusted for age (y), sex (male or female), and BMI (kg/m^2^). Model 2 was further adjusted for marital status (yes or no), household income (≤3000, 3000–6000 or >6000 yuan·month^−1^·person^−1^), smoking status (yes or no), drinking status (yes or no), physical activity (Met/week), calcium supplement use (yes or no), multivitamin use (yes or no), dietary energy intake (kcal/d), dietary vitamin D intake (μg/d), and dietary calcium intake (mg/d). Protein intakes were energy-adjusted residuals added to a constant, where the constant equals the nutrient intake for the mean energy intake of the study population. Changes of BMD = [(BMD _follow-up 2_ − BMD _follow-up 1_) × 3]/time interval. % Difference = (Q4 − Q1)/ABS (Q1) × 100%.

**Table 4:** Changes in bone mineral density (BMD) (mg/cm^2^) between two follow-ups by quartiles (Q) of animal protein intake (n = 1987).

	Dietary Animal Protein Intakes (g·kg^−1^·d^−1^)						
	Q1 (<0.42)	Q2 (0.42~)	Q3 (0.54~)	Q4 (≥0.68)	% Difference	*p*-ANCOVA	*p* Trend
	n = 497	n = 496	n = 497	n = 497			
BMD changes, mg/cm^2^							
Whole body							
Model 1	−286.02 ± 7.77	−294.29 ± 7.49	−289.75 ± 7.45	−284.70 ± 7.80	0.42	0.805	0.815
Model 2	−284.21 ± 8.01	−294.30 ± 7.57	−290.71 ± 7.55	−285.95 ± 8.05	−0.61	0.775	0.966
Spine L1-4							
Model 1	280.64 ± 7.18	292.41 ± 6.92	285.39 ± 6.88	275.36 ± 7.20	−1.88	0.359	0.496
Model 2	279.47 ± 7.37	292.67 ± 6.97	285.71 ± 6.94	275.03 ± 7.41	−1.59	0.312	0.565
Total hip							
Model 1	−22.10 ± 11.38	−13.38 ± 11.01	−3.36 ± 10.95	−2.09 ± 11.48	90.54	0.594	0.189
Model 2	−24.40 ± 11.70	−14.75 ± 11.11	−2.91 ± 11.05	−1.43 ± 11.83	94.14	0.514	0.151
Femur neck							
Model 1	−429.57 ± 7.51	−443.97 ± 7.24	−424.13 ± 7.20	−419.15 ± 7.54	2.43	0.099	0.145
Model 2	−431.04 ± 7.72	−445.04 ± 7.29	−422.44 ± 7.27	−417.33 ± 7.75	3.18	0.057	0.084
Trochanter							
Model 1	−15.58 ± 1.84	−15.96 ± 1.77	−13.43 ± 1.76	−13.07 ± 1.84	16.11	0.604	0.241
Model 2	−15.24 ± 1.89	−16.12 ± 1.78	−13.11 ± 1.78	−13.50 ± 1.90	11.42	0.633	0.363

Data are presented as the adjusted mean ± standard error based on the general linear model with the Q1 as the reference group. Model 1 was adjusted for age (y), sex (male or female), and BMI (kg/m^2^). Model 2 was further adjusted for marital status (yes or no), household income (≤3000, 3000–6000 or >6000 yuan·month^−1^·person^−1^), smoking status (yes or no), drinking status (yes or no), physical activity (Met/week), calcium supplement use (yes or no), multivitamin use (yes or no), dietary energy intake (kcal/d), dietary vitamin D intake (μg/d), and dietary calcium intake (mg/d). Protein intakes were energy-adjusted residuals added to a constant, where the constant equals the nutrient intake for the mean energy intake of the study population. Changes in BMD = [(BMD _follow-up 2_ − BMD _follow-up 1_) × 3]/time interval. % Difference = (Q4 − Q1)/ABS (Q1) × 100%.

**Table 5:** Changes in bone mineral density (BMD) (mg/cm^2^) between two follow-ups by quartiles (Q) of white meat protein intake (n = 1987).

	Dietary White Meat Protein Intakes (g·kg^−1^·d^−1^)						
	Q1 (<0.12)	Q2(0.12~)	Q3(0.17~)	Q4(≥0.26)	% Difference	*p*-ANCOVA	*p* Trend
	n = 496	n = 498	n = 496	n = 497			
BMD changes, mg/cm^2^							
Whole body							
Model 1	−287.55 ± 7.59	−291.38 ± 7.49	−288.93 ± 7.45	−286.94 ± 7.56	0.21	0.976	0.901
Model 2	−287.76 ± 7.68	−290.91 ± 7.58	−289.19 ± 7.53	−287.34 ± 7.69	0.15	0.987	0.933
Spine L1-4							
Model 1	280.54 ± 7.01	283.91 ± 6.92	291.38 ± 6.89	278.02 ± 6.98	−0.90	0.547	0.998
Model 2	281.34 ± 7.07	282.96 ± 6.97	291.50 ± 6.93	277.17 ± 7.08	−1.48	0.520	0.902
Total hip							
Model 1	−14.70 ± 11.14	−15.88 ± 10.97	−14.49 ± 10.96	3.99 ± 11.12	127.14	0.537	0.255
Model 2	−16.24 ± 11.23	−16.74 ± 11.23	−15.26 ± 11.06	4.49 ± 11.28	127.65	0.486	0.218
Femur neck							
Model 1	−443.62 ± 7.33	−429.37 ± 7.24	−428.91 ± 7.20	−415.19 ± 7.30 *	6.41	0.062	0.010
Model 2	−445.60 ± 7.39	−429.92 ± 7.29	−426.95 ± 7.25	−413.68 ± 7.40 *	7.16	0.030	0.004
Trochanter							
Model 1	−16.99 ± 1.79	−16.38 ± 1.77	−13.07 ± 1.76	−11.66 ± 1.78	31.37	0.107	0.017
Model 2	−17.08 ± 1.81	−16.28 ± 1.78	−12.92 ± 1.77	−11.75 ± 1.81	31.21	0.122	0.020

Data are presented as the adjusted mean ± standard error based on the general linear model with Q1 as the reference group. * *p* < 0.05. White meat protein included meat protein from poultry, fish, and seafoods. Model 1 was adjusted for age (y), sex (male or female), and BMI (kg/m^2^). Model 2 was further adjusted for marital status (yes or no), household income (≤3000, 3000-6000 or >6000 yuan·month^−1^·person^−1^), smoking status (yes or no), drinking status (yes or no), physical activity (Met/week), calcium supplement use (yes or no), multivitamin use (yes or no), dietary energy intake (kcal/d), dietary vitamin D intake (μg/d), and dietary calcium intake (mg/d). Protein intakes were energy-adjusted residuals added to a constant, where the constant equals the nutrient intake for the mean energy intake of the study population. Changes in BMD = [(BMD _follow-up 2_ − BMD _follow-up 1_) × 3]/time interval. % Difference = (Q4 − Q1)/ABS (Q1) × 100%.

**Table 6:** Associations of dietary amino acids intakes with changes of bone mineral density (BMD) (mg/cm^2^) (n = 1987).

Dietary Amino Acids Intakes (mg·kg^−1^d^−1^)	Whole Body	Spine L1-4	Total Hip	Femur Neck	Trochanter
Lysine	0.03 ± 0.25	0.04 ± 0.23	0.36 ± 0.36	0.44 ± 0.24	0.09 ± 0.06
Tryptophan	−0.68 ± 1.44	1.37 ± 1.33	2.58 ± 2.10	1.95 ± 1.39	0.68 ± 0.34 *
Phenylalanine	−0.33 ± 0.44	0.46 ± 0.40	0.82 ± 0.64	0.85 ± 0.42 *	0.25 ± 0.10 *
Threonine	−0.09 ± 0.46	0.22 ± 0.42	0.65 ± 0.67	0.90 ± 0.44 *	0.19 ± 0.11
Isoleucine	0.01 ± 0.44	0.19 ± 0.41	0.73 ± 0.65	0.94 ± 0.43 *	0.21 ± 0.10 *
Leucine	−0.06 ± 0.24	0.15 ± 0.22	0.37 ± 0.36	0.49 ± 0.23 *	0.12 ± 0.06 *
Valine	−0.18 ± 0.40	0.27 ± 0.36	0.63 ± 0.58	0.83 ± 0.38 *	0.21 ± 0.09 *
Histidine	−0.20 ± 0.67	0.36 ± 0.62	0.77 ± 0.98	1.22 ± 0.65	0.25 ± 0.16
Methionine	−0.23 ± 0.82	0.38 ± 0.75	1.05 ± 1.20	1.59 ± 0.79 *	0.31 ± 0.19
Cystine	0.35 ± 1.35	−0.06 ± 1.24	3.04 ± 1.98	3.82 ± 1.30 **	0.51 ± 0.32
Sulfur amino acids	−0.12 ± 0.55	0.25 ± 0.50	0.90 ± 0.80	1.17 ± 0.53 *	0.22 ± 0.13

Data are presented as regression coefficient ± standard error. The analyses were conducted via multivariable linear regression by means of the enter method with the adjusted covariates including age (y), sex (male or female), BMI (kg/m^2^), marital status (yes or no), household income (≤3000, 3000–6000 or >6000 yuan·month^−1^·person^−1^), smoking status (yes or no), drinking status (yes or no), total physical activity (Met/week), calcium supplement usage (yes or no), multivitamin usage (yes or no), dietary energy intake (kcal/d), dietary vitamin D intake (ug/d), and calcium intake (mg/d). Changes in BMD = [(BMD _follow-up 2_ − BMD _follow-up 1_)×3]/time interval. Dietary amino acids intakes were estimated on the basis of the validated food frequency questionnaire and the Chinese Food Composition Table 2009. * *p* < 0.05, ** *p* < 0.01.

## Data Availability

Not applicable.

## Supplementary Materials

The following supporting information can be downloaded at: https://www.mdpi.com/article/10.3390/nu15061432/s1, Table S1: Dietary amino acids intake at 1st follow-up (2011.04 to 2013.03) by quartiles of total protein intakes, Guangzhou Nutrition and Health Cohort (n = 1987). Table S2: Changes of BMD (mg/cm^2^) by quartiles of dietary plant protein intakes. Table S3: Subgroup analyses by gender for the associations of dietary total protein intakes (g·kg^−1^·d^−1^) with the changes of BMD (mg/cm^2^) by multivariable linear regression (n = 1987). Table S4: Subgroup analyses by age (≥ vs. < 60y) for the associations of dietary total protein intake (g·kg^−1^·d^−1^) and BMD changes (mg/cm^2^) by multivariable linear regression. Table S5: Subgroup analyses by Body mass index (BMI, ≥24.0 vs. <24.0 kg/m^2^) for the associations of various dietary protein intake with the changes of BMD (mg/cm^2^) by multivariable linear regression. Table S6: Subgroup analyses by high and low levels of dietary calcium intake for the associations of various dietary protein intake with the changes of BMD (mg/cm^2^) by multivariable linear regression analyses. Table S7: Replacing red meat protein with white meat protein by multivariable-adjusted substitution analyses.
