# Supplementary material for: Increased Dietary Intakes of Total Protein, Animal Protein and White Meat Protein Were Associated with Reduced Bone Loss—A Prospective Analysis Based on Guangzhou Health and Nutrition Cohort, South China
Source: Nutrients. 2023 Mar 16;15(6):1432. doi: 10.3390/nu15061432 (PMC10051092; doi:10.3390/nu15061432)
Supplement: Supplementary file 1 [file nutrients-15-01432-s001.zip › nutrients-2257763-supplementary.pdf]

Supplemental Tables

Supplemental Table S1. Dietary amino acids intake at 1st follow-up (2011 to 2013) by quartiles of total protein intakes, Guangzhou Nutrition and Health Study (n=1987).

Dietary amino acids intakes, mg·kg^{-1}·d^{-1}	Dietary total protein intakes (g·kg^{-1}·d^{-1})					
	Q1(<0.96)	Q2(0.96~)	Q3(1.10~)	Q4(≥1.26)	Total	*P*
n	497	497	497	496	1987	
Isoleucine	37.51±7.09	42.85±8.07	46.62±9.16	53.24±11.13	45.05±10.65	< 0.001
Leucine	70.09±12.89	80.08±14.68	87.03±16.61	99.42±20.27	84.15±19.51	< 0.001
Lysine	54.84±12.31	62.96±14.03	68.75±16.18	78.88±19.43	66.35±17.98	< 0.001
Methionine	19.47±3.81	22.20±4.29	24.26±5.18	27.59±5.89	23.38±5.69	< 0.001
Phenylalanine	43.46±7.29	49.51±8.72	53.42±9.34	60.94±11.49	51.83±11.28	< 0.001
Threonine	35.83±6.85	40.99±7.71	44.58±8.87	50.92±10.80	43.08±10.27	< 0.001
Valine	46.84±8.12	55.34±9.21	57.77±10.50	65.90±12.78	55.96±12.41	< 0.001
Tryptophan	11.20±2.11	12.85±2.74	13.92±2.76	15.85±3.29	13.45±3.23	< 0.001
Histidine	23.14±4.54	26.50±5.23	28.87±5.78	33.04±7.33	27.88±6.84	< 0.001
Cystine	13.60±2.36	15.48±3.02	16.76±3.03	19.06±3.57	16.22±3.62	< 0.001
Sulfur Amino Acids	32.38±5.82	36.92±6.87	40.14±7.73	45.62±8.89	38.76±8.84	< 0.001

Data were presented as mean ± SD for continuous variables and compared by Analysis of variance (ANOVA). Dietary amino acids intakes were estimated based on the Food Frequency Questionnaire and Chinese Food Composition Table. They were residual adjusted by adding residuals to the mean energy intake of participants. Sulfur Amino Acids= Cystine+ Methionine

Supplemental Table S2. Changes of BMD (mg/cm^2) by quartiles of dietary plant protein intakes.

	Dietary plant protein intakes ($g\cdot kg^{-1}\cdot d^{-1}$)						
	Q1(<0.47) n=496	Q2 (0.47~) n=497	Q3 (0.56~) n=497	Q4(≥0.64) n=497	% difference	*P*-ANCOVA	*P* trend
BMD changes, mg/cm^2							
Whole body							
Model I	-292.67±7.89	-282.48±7.48	-285.97±7.45	-293.79±8.08	-0.38	0.669	0.856
Model II	-290.37±8.39	-281.97±7.58	-287.45±7.53	-295.48±8.39	-1.76	0.667	0.612
Spine L1-4							
Model I	286.29±7.29	276.80±6.91	283.28±6.89	287.57±7.47	0.45	0.698	0.768
Model II	284.01±7.73	275.53±6.98	285.03±6.93	288.37±7.73	1.54	0.629	0.549
Total hip							
Model I	0.19±11.60	-11.98±10.98	-21.95±10.94	-7.15±11.88	-3863.16	0.553	0.565
Model II	-0.97±12.30	-13.03±11.11	-20.87±11.04	-8.59±12.30	-785.57	0.665	0.609
Femur neck							
Model I	-436.68±7.64	-425.40±7.24	-425.44±7.22	-429.28±7.83	1.72	0.675	0.545
Model II	-437.53±8.10	-425.19±7.31	-424.44±7.26	-428.69±8.10	2.02	0.621	0.490
Trochanter							
Model I	-16.24±1.86	-14.13±1.77	-12.81±1.76	-14.86±1.91	8.50	0.593	0.541
Model II	-17.61±1.98	-14.06±1.79	-12.38±1.77	-13.93±1.98	20.90	0.284	0.188

Data were presented as the adjusted mean ± standard error by General Linear Model with Q1 as reference group.

Model I was adjusted for age (y), sex (male or female) and BMI (kg/m^2). Model Ⅱ was further adjusted for marital status (yes or no), household income (≤3000, 3000-6000 or >6000 yuan • m^{-1} • p^{-1}), smoking status (yes or no), drinking status (yes or no), physical activity (Met • wk^{-1}), calcium supplement use

(yes or no), multi-vitamins use (yes or no), dietary energy intake (kcal/d), dietary Vitamin D intake (μg/d) and dietary calcium intake (mg/d).

Protein intakes were energy-adjusted residuals added to a constant, where the constant equals the nutrient intake for the mean energy intake of the study population. Changes of BMD =[(BMD at 2nd follow-up – BMD at 1st follow- up)×3] / time interval.   % Difference = (Q4 − Q1) / ABS (Q1) * 100%

Supplemental Table S3. Subgroup analyses by gender for the associations of dietary total protein intakes ($g \cdot kg^{-1} \cdot d^{-1}$) with the changes of BMD (mg/cm^2) by multivariable linear regression (n=1987).

Dietary protein intakes ($g \cdot kg^{-1} \cdot d^{-1}$)	Changes of bone mineral density at whole body and different sites (mg/cm^2)				
	Whole body	Spine L1-4	Total hip	Femur neck	Trochanter
**Men (*n*=563)**					
Total protein	-17.65±44.02	2.33±39.20	55.40±61.60	43.90±41.06	15.03±7.32*
Animal protein	-24.85±42.52	-2.41±37.87	19.10±59.70	20.18±39.70	6.10±7.09
Red-meat protein	-100.49±59.72	49.69±53.27	-55.94±83.80	7.90±55.90	-7.17±9.99
White-meat protein	66.40±65.38	-48.29±58.24	151.76±92.66	33.04±61.08	19.84±10.89
Milk and dairy protein	-5.73±210.04	-88.41±186.98	-322.27±293.20	287.63±195.69	15.37±35.04
Egg protein	-52.43±220.02	-92.58±195.88	-49.86±307.80	-255.72±205.11	19.09±36.70
Plant protein	23.37±71.03	12.80±63.25	91.20±99.39	57.99±66.27	22.11±11.81
Soy protein	57.18±105.46	-92.48±93.84	-12.57±147.51	88.34±98.41	30.21±17.55
**Women (*n*=1424)**					
Total protein	-0.48±23.46	11.01±21.71	31.52±35.01	75.91±22.83***	20.08±6.15**
Animal protein	22.28±22.62	-5.26±20.95	40.92±33.76	63.22±22.05**	14.83±5.94*
Red-meat protein	36.49±35.31	1.28±32.69	65.95±52.76	17.71±34.51	11.76±9.29
White-meat protein	4.31±33.69	-15.53±31.18	34.81±50.28	102.69±32.80**	19.73±8.85*
Milk and dairy protein	93.14±86.98	-32.83±80.53	-24.76±129.77	66.82±85.00	21.49±22.90
Egg protein	-15.83±92.64	57.61±85.72	-1.95±138.29	85.35±90.48	-5.89±24.38
Plant protein	-58.86±36.38	40.14±33.69	-30.04±54.39	19.17±35.58	9.96±9.58
Soy protein	-103.48±60.49	67.99±56.01	125.94±90.37	-84.08±59.12	-3.73±15.94

Data were presented as regression coefficients ± standard error (B±SE) by multivariable linear regression. Model was adjusted for age (y), BMI (kg/m^2),

marital status (yes or no), household income (≤3000, 3000-6000 or >6000 yuan·m-1·p-1), smoking (yes or no), alcohol drinking (yes or no), total physical activity (Met·wk^{-1}), calcium supplement usage (yes or no), multi-vitamin usage (yes or no), dietary energy intake (kcal/d), dietary Vitamin D intake (ug/d) and calcium intake (mg/d). Changes of BMD = [(BMD at 2nd follow-up – BMD at 1st follow- up)×3] / time interval. Additional correction of estrogen usage was made for women. $^{*}p < 0.05$, $^{**}p < 0.01$, $^{***}p < 0.001$

Supplemental Table S4. Subgroup analyses by age (≥ vs. < 60y) for the associations of dietary total protein intake ($g·kg^{-1}·d^{-1}$) and BMD changes (mg/cm^2) by multivariable linear regression.

Dietary protein intakes ($g·kg^{-1}·d^{-1}$)	Changes of bone mineral density at whole body and different sites (mg/cm^2)				
	Whole body	Spine L1-4	Total hip	Femur neck	Trochanter
**≥60 y (*n*=876)**					
Total protein	30.55±36.62	-20.75±31.23	-5.56±46.82	65.50±30.23*	13.27±6.09*
Animal protein	56.08±34.92	-35.76±29.79	5.56±44.70	56.11±28.87*	8.09±5.82
Red-meat protein	31.69±52.05	1.61±44.40	-13.37±66.60	42.04±43.06	3.08±8.67
White-meat protein	81.02±54.82	-92.12±46.70*	41.33±70.48	98.05±45.30*	13.83±9.13
Milk and dairy protein	195.76±147.88	-9.74±126.23	-89.21±188.76	38.09±122.48	24.75±24.65
Egg protein	-36.25±148.26	25.95±126.42	-0.52±189.28	-85.82±122.65	-5.30±24.70
Plant protein	-66.24±53.51	39.52±45.64	-24.97±68.49	8.37±44.31	9.38±8.92
Soy protein	-125.05±80.78	50.43±68.96	69.70±103.40	-58.59±66.91	4.19±13.48
**<60 y (*n*=1022)**					
Total protein	-25.36±23.40	25.72±23.70	58.85±40.68	67.60±27.08*	23.86±7.45**
Animal protein	-28.37±22.73	11.95±23.04	51.56±39.57	48.44±26.35	15.32±7.26*
Red-meat protein	-36.55±34.99	15.97±35.45	62.61±60.93	-8.72±40.61	7.50±11.19
White-meat protein	-21.17±32.98	9.21±33.41	66.29±57.50	80.67±38.18*	23.99±10.52*
Milk and dairy protein	-35.06±87.52	-12.40±88.64	-78.18±152.10	113.66±101.47	5.33±27.97
Egg protein	-9.56±99.67	31.97±100.94	-18.70±173.34	116.52±115.56	7.37±31.86
Plant protein	12.67±38.48	35.29±38.95	11.71±66.94	44.03±44.61	20.63±12.28
Soy protein	85.88±66.97	-27.23±67.87	137.16±116.39	12.94±77.75	15.92±21.42

Data were presented as regression coefficients ± standard errors by multivariable linear regression by enter method. Model was adjusted for sex (male or

female), BMI (kg/m^2), marital status (yes or no), household income (≤3000, 3000-6000 or >6000 yuan·m^{-1}·p^{-1}), smoking (yes or no), alcohol drinking (yes or no), total physical activity (Met·wk^{-1}), calcium supplement usage (yes or no), multi-vitamin usage (yes or no), daily dietary energy intake (kcal/d), dietary Vitamin D intake (ug/d) and calcium intake (mg/d). Changes of BMD =[(BMD at 2nd follow-up – BMD at 1st follow- up)×3] / time interval. $^{*}p < 0.05$, $^{**}p < 0.01$, $^{***}p < 0.001$

Supplemental Table S5. Subgroup analyses by Body mass index (BMI, ≥24.0 vs. <24.0 kg/m²) for the associations of various dietary protein intake with the changes of BMD (mg/cm²) by multivariable linear regression.

Dietary protein intakes	Changes of bone mineral density at whole body and different sites (mg/cm²)				
($g \cdot kg^{-1} \cdot d^{-1}$)	Whole body	Spine L1-4	Total hip	Femur neck	Trochanter
**BMI≥24.0 kg/m²（n=826）**					
Total protein	18.98±36.29	-32.98±32.52	32.25±48.78	47.46±33.33	7.30±7.42
Animal protein	44.85±35.21	-30.58±31.58	26.41±47.28	40.69±32.37	3.39±7.21
Red-meat protein	91.25±55.25	-4.97±49.62	-5.21±74.25	13.62±50.87	-1.62±11.32
White-meat protein	-1.19±56.16	-82.16±50.26	90.32±75.29	88.29±51.53	11.13±11.48
Milk and dairy protein	134.48±155.95	72.62±139.85	69.94±209.24	60.34±143.41	4.26±31.92
Egg protein	35.27±173.88	2.02±155.89	-258.50±233.12	-65.38±159.83	-13.49±35.57
Plant protein	-69.00±56.33	-1.29±50.55	9.93±75.61	10.38±51.83	8.94±11.53
Soy protein	-173.32±98.09	37.75±88.11	37.80±132.11	-48.10±90.34	-3.54±20.11
**18.5≤BMI<24.0kg/m²（*n*=1083）**					
Total protein	-48.57±24.11*	61.42±22.80**	66.99±38.75	70.78±24.64**	17.27±6.42**
Animal protein	-40.04±24.98	35.51±23.66	77.80±40.19	53.85±25.56*	12.47±6.66
Red-meat protein	-95.48±38.11*	63.30±36.14	68.08±61.58	-18.82±39.14	2.76±10.20
White-meat protein	8.57±36.13	16.39±34.21	91.92±58.25	95.92±36.87**	21.75±9.61*
Milk and dairy protein	-68.62±97.19	18.19±92.05	-20.54±156.00	129.77±99.45	16.02±25.93
Egg protein	20.27±99.32	-10.25±94.04	98.93±159.53	115.65±101.63	-3.03±26.49
Plant protein	-28.32±38.38	71.39±36.28	-13.40±61.75	51.86±39.27	14.21±10.23
Soy protein	49.76±63.05	-29.19±59.71	61.39±101.26	2.37±64.58	8.96±16.82

Data were presented as regression coefficients ± standard errors by multivariable linear regression. Model was adjusted for age (y), sex (male or female), marital status (yes or no), household income (≤3000, 3000-6000 or >6000 yuan·m^{-1}·p^{-1}), smoking (yes or no), alcohol drinking (yes or no), total physical activity (Met·wk^{-1}), calcium supplement usage (yes or no), multi-vitamin usage (yes or no), daily dietary energy intake (kcal/d), dietary Vitamin D intake (ug/d) and calcium intake (mg/d). Changes of BMD =[(BMD at 2nd follow-up – BMD at 1st follow- up)×3] / time interval. $^{*}p < 0.05$, $^{**}p < 0.01$, $^{***}p < 0.001$

Supplemental Table S6. Subgroup analyses by high and low levels of dietary calcium intake for the associations of various dietary protein intake with the changes of BMD (mg/cm^2) by multivariable linear regression analyses.

Dietary protein intakes	Changes of bone mineral density at whole body and different sites (mg/cm^2)				
(g·kg^{-1}·d^{-1})	Whole body	Spine L1-4	Total hip	Femur neck	Trochanter
**≥50.00% RNI (*n*=958)**					
Total protein	8.10±30.39	1.28±26.92	-9.56±43.07	70.18±27.24**	16.67±6.04**
Animal protein	37.93±28.85	-17.36±25.57	0.00±40.93	67.21±25.87**	10.86±5.75
Red-meat protein	2.46±46.10	32.83±40.81	-29.49±65.43	-22.62±41.45	11.91±9.19
White-meat protein	38.81±39.31	-48.93±34.80	22.76±56.00	91.79±35.25**	9.25±7.84
Milk and dairy protein	93.08±77.87	-29.92±69.02	-9.27±110.42	125.65±69.97	17.20±15.54
Egg protein	58.49±121.97	4.09±108.04	11.68±172.96	167.33±109.58	-20.79±24.32
Plant protein	-75.55±45.28	45.69±40.14	-21.33±64.33	-9.56±40.79	10.34±9.04
Soy protein	-85.78±64.13	61.73±56.82	102.78±90.98	-25.01±57.73	1.40±12.81
**<50.00%RNI (*N*=1029)**					
Total protein	-2.46±28.20	11.87±26.92	67.89±42.49	68.00±28.88*	19.61±7.51**
Animal protein	-7.12±27.88	2.71±26.62	67.98±42.04	40.42±28.61	13.01±7.44
Red-meat protein	-13.00±40.29	0.80±38.47	56.76±60.76	44.43±41.36	1.43±10.77
White-meat protein	9.02±48.24	0.74±46.06	137.15±72.63	90.77±49.46	37.91±12.84**
Milk and dairy protein	-0.18±147.44	-21.19±140.76	-50.41±222.13	-110.59±151.39	-28.58±39.41
Egg protein	-78.63±125.13	57.30±119.48	-67.25±188.70	-145.55±128.46	13.73±33.46
Plant protein	13.48±47.13	25.40±45.00	-4.26±71.16	74.46±48.35	17.61±12.59
Soy protein	59.11±93.98	-73.84±89.71	66.52±141.81	-22.97±96.54	23.96±25.12

Data were presented as regression coefficients ± standard errors by multivariable linear regression. Model was adjusted for age (y), sex (male or female), BMI

(kg/m^2), marital status (yes or no), household income (≤3000, 3000-6000 or >6000 yuan·m^{-1}·p^{-1}), smoking (yes or no), alcohol drinking (yes or no), total physical activity (Met·wk^{-1}), calcium supplement usage (yes or no), multi-vitamin usage (yes or no), dietary energy intake (kcal/d), dietary Vitamin D intake (ug/d). Changes of BMD =[(BMD at 2nd follow-up – BMD at 1st follow- up)×3] / time interval. RNI, recommended nutrients intake by Chinese Nutrition Society; $^{*}p < 0.05$, $^{**}p < 0.01$, $^{***}p < 0.001$

Supplemental Table S7. Replacing red meat protein with white meat protein by multivariable-adjusted substitution analyses.

Replacement of red meat protein intake with white meat protein intake, $g \cdot kg^{-1} \cdot d^{-1}$	Changes of bone mineral density at different sites (mg/cm^2)									
	Whole body		Spine L1-4		Total hip		Femur neck		Trochanter	
	B±SE	β	B±SE	β	B±SE	β	B±SE	β	B±SE	β
Original model (no substitution)										
Model I	22.93±40.32	0.018	-40.46±37.11	-0.035	55.34±59.17	0.030	64.49±38.80	0.053	13.89±9.48	0.047
Leave-one-out model										
Model II (substation model)	33.67±44.62	0.027	-47.28±41.07	-0.041	35.01±65.43	0.019	82.86±42.92	0.069	15.87±10.49	0.054

Model I was the original multivariable adjusted linear regression model. The variables included in the models were white meat protein intake ($g \cdot kg^{-1} \cdot d^{-1}$), animal protein intake ($g \cdot kg^{-1} \cdot d^{-1}$), , age (y), sex (male or female), BMI (kg/m2), marital status (yes or no), household income (≤3000, 3000-6000 or >6000 yuan·m-1·p-1), smoking (yes or no), alcohol drinking (yes or no), total physical activity (Met·wk-1), calcium supplement usage (yes or no), multi-vitamin usage (yes or no), daily dietary energy intake (kcal/d), dietary Vitamin D intake (μg/d) and calcium intake (mg/d).

Model II was the model used for substitution analyses by Leave-one-out method which included an additional variable (the other sources of animal protein, g·kg-1·d-1, protein from egg and dairy etc.) on the basis of Model I.

B denoted the coefficient of white meat protein intake($g \cdot kg^{-1} \cdot d^{-1}$) in multivariable linear regression model; SE denoted standard error; β was the standardized coefficient for white meat protein intake($g \cdot kg^{-1} \cdot d^{-1}$) in multivariable linear regression model.
